# Antibodies against integrin αvβ6 have high diagnostic accuracy for ulcerative colitis

**DOI:** 10.3389/fimmu.2025.1641329

**Published:** 2025-08-21

**Authors:** Patrick Bez, Martina Scapolan, Davide Giuseppe Ribaldone, Gian Paolo Caviglia, Silvia Zago, Cristina Trucco, Simone Frara, Antonino Caruso, Marta Ascolani, Michele Campigotto, Stefano Benvenuti, Stefano Martelossi, Lara De Lucchi, Marta Favero, Francesco Cinetto, Elisabetta Faggin, Laura Ventura, Marcello Rattazzi, Tilde Manetta, Giulio Mengozzi, Antonio Antico, Carla Felice

**Affiliations:** ^1^ Medicine 1 Unit, Ca’ Foncello University Hospital, Treviso, Italy; ^2^ Department of Laboratory Medicine, Ca’ Foncello University Hospital, Treviso, Italy; ^3^ Department of Medical Sciences, University of Turin, Turin, Italy; ^4^ Gastroenterology Unit, Ca’ Foncello University Hospital, Treviso, Italy; ^5^ Pediatric Unit, Ca’ Foncello University Hospital, Treviso, Italy; ^6^ Department of Medicine (DIMED), University of Padova, Padova, Italy; ^7^ Department of Statistical Sciences, University of Padova, Padova, Italy; ^8^ Department of Laboratory Medicine, Città della Salute e della Scienza - Molinette Hospital, Turin, Italy

**Keywords:** ulcerative colitis, Crohn’s disease, integrin αvβ6, diagnosis, inflammatory bowel disease

## Abstract

**Background:**

Anti-integrin αvβ6 IgG autoantibodies showed good sensitivity and optimal specificity in ulcerative colitis (UC) compared to controls. We aim at confirming the diagnostic accuracy of anti-integrin αvβ6 autoantibodies in an Italian multicentric cohort.

**Methods:**

This observational multicentric study included adult and pediatric patients with inflammatory bowel disease and controls. Data on demographics, disease extension, partial Mayo score, fecal calprotectin, endoscopic Mayo score, and the time to the composite outcome including hospitalization or colectomy were collected. A new commercial ELISA kit was used to measure anti-integrin αvβ6 in the serum of the enrolled patients. Receiver operating curve (ROC) was used to identify the optimal cutoff to discriminate UC cases from other patients. Kaplan–Meier curves and log-rank test were used to analyze the composite outcome hospitalization and need of colectomy.

**Results:**

A total of 228 patients were enrolled, including 36 controls (13 healthy donors and 24 diseased controls), 34 irritable bowel syndrome (IBS) patients, 50 Crohn’s disease (CD) patients, and 107 UC patients. The UC patients presented higher values of anti-integrin αvβ6 IgG compared to CD, IBS, and controls (Kruskal–Wallis test and *post-hoc* Holm’s correction: *p* < 0.001). The ROC of anti-integrin αvβ6 IgG performed optimally with an area under the curve of 0.93. The optimal cutoff to distinguish UC from controls was 1.68 U/mL, with a sensitivity of 87.9% and a specificity of 86.8% compared to non-UC patients with a specificity of 94.4% to non-IBD and 76% to CD, with very similar values to a recent multicentric study. A higher threshold up to 13 U/mL may be useful to make a differential diagnosis between UC and CD with a specificity of 90%. Anti-integrin αvβ6 did not correlate with clinical disease activity but weakly with fecal calprotectin (*R* = 0.28, *p* = 0.36) and moderately with endoscopic disease activity reported at the last colonoscopy (*R* = 0.60, *p* = 0.03). Despite the low number of events, the log-rank test showed the potential predictive performance of high levels of anti-integrin αvβ6 IgG (i.e., >17 U/mL) for the composite outcome (*p* = 0.02).

**Conclusions:**

This study validates a new anti-integrin αvβ6 ELISA kit and confirms its high diagnostic accuracy in UC also in a European population, with particular utility in the differential diagnosis of specific forms of IBD.

## 1 Introduction

Inflammatory bowel disease (IBD) is a chronic disorder of the gut and includes two main entities: ulcerative colitis (UC) and Crohn’s disease (CD). Their pathogenesis results from the combination of genetic, environmental, and immune factors. UC is the most frequent IBD, and an autoimmune component in the pathogenesis has been postulated (1). A continuous, diffuse, and circumferential pattern of inflammation in colonic mucosa and submucosa represents the typical endoscopic presentation, usually affecting the rectum with a variable proximal extension up to the ileocecal valve; the involvement of the last tract of ileum (“backwash ileitis”) is also possible (2). CD may involve any section of the gastrointestinal tract, with prevalent location to the terminal ileum, and presents a typically segmental mucosa involvement. The ECCO-ESGAR guidelines indicate that ileo-colonoscopy with biopsies is the preferred method to confirm the diagnosis of IBD, differentiating between UC and CD, and to assess disease activity (3). In a minority of cases with exclusive colonic involvement, the diagnosis of a specific type of IBD may still remain undefined or reclassified over time (4). Thus, the discovery of sensible, reliable, and economic serological markers that support the diagnosis of UC over CD or vice versa is warranted to aid the differential diagnosis in this particular setting. The best serological markers currently available in the clinical practice for the differential diagnosis of IBD are ASCA (anti-*Saccharomyces* antibodies) for CD and the atypical p-ANCA for UC (5). However, the ECCO-ESGAR guidelines do not recommend them for routine use considering their low accuracy (3). In 2021, Kuwada et al. described the presence of anti-integrin αvβ6 IgG autoantibodies in the sera of Japanese patients with UC (6). Shortly after, the prevalence of anti-integrin αvβ6 IgG autoantibodies was described in several cohorts around the world (7–10). These autoantibodies are mainly of the IgG1 class and inhibit integrin-αvβ6 fibronectin binding. This integrin is expressed in the colonic mucosa but not in the small intestine (6). Most of the previous literature on anti-integrin αvβ6 IgG autoantibodies was based on homemade ELISA kit, which is more time-consuming and not suitable for routine diagnostic. Here, we aim at confirming the diagnostic accuracy of anti-integrin αvβ6 IgG autoantibodies in an Italian multicentric cohort of IBD patients, investigating also their potential associations with UC clinical activity and extension and validating a new commercial ELISA kit for absolute quantification of anti-integrin αvβ6 IgG autoantibodies also in the European population. Our results confirm the optimal performance of the test in discriminating UC from controls. For the first time, a higher threshold to increase the specificity of UC over CD is proposed to increase diagnostic performance in case the endoscopy and histology do not allow the discrimination of the two entities. In the future, integration of anti-integrin αvβ6 IgG with endoscopic features may allow the reclassification of most IBD-U cases.

## 2 Methods

This is an Italian multicentric study including patients referred to the University Hospital Ca’ Foncello of Treviso (TV) and the Hospital Molinette of Torino (TO), two tertiary referral centers for the treatment of IBD. The study was approved by the Ethics Committee of Treviso (CET ANV 2024-56) and University of Torino (0056924) and complies with the Helsinki Declaration of 1964.

### 2.1 Patients’ enrollment

In Treviso, sera were collected for the purpose of this study from consecutive IBD patients during routine outpatient clinic visits in October and November 2024. Additional sera previously collected for research purposes in 2021 (793/CE Marca Trevigiana and 366/CE Marca Trevigiana) and including IBD patients and controls were used for this analysis. In Torino, sera from UC and irritable bowel syndrome (IBS) patients were collected from March 2017 to October 2023; the sera of UC patients were harvested before starting a new therapy. All patients have signed an informed consent to participate in the study.

### 2.2 Data collection

The patients’ data were collected at the time of sampling, integrated from medical records, as needed, and anonymously recorded in a shared Excel dataset. For all patients, data on demographics, smoking status, disease type and localization/extension according to Montreal classification, and disease duration were collected. In UC patients, clinical activity was also measured using the partial Mayo score (PMS), and treatments ongoing at the time of sampling were specified. The values of fecal calprotectin (FCP) and endoscopic activity according to the Mayo endoscopic score, if available, were considered only if performed within the last 60 and 90 days before sampling, respectively. For UC patients with a follow-up of at least of 1 year after the sampling, the composite outcome of hospitalization and need for colectomy was calculated until the end of December 2024.

### 2.3 Sampling and storage

Blood samples were collected. Serum was obtained within 1 h from blood withdrawal and stored at -80°C until the analysis.

### 2.4 ELISA technique

The anti-integrin αvβ6 IgG was tested using the “anti-integrin αvβ6 ELISA kit” (Medical and Biological Laboratories Co., Ltd; JSR Life Sciences Company), provided by the company MBL (Medical and Biological Laboratories Co., Ltd; JSR Life Sciences Company). The analysis was performed according to the manufacturer’s instruction by the same technician at both institutions. The intra-assay reproducibility was obtained by measuring nine samples eight times with coefficient of variation (CV) of less than 10%. The limit of quantification (LoQ) is considered to be 0.4 U/mL.

### 2.5 Statistical analysis

The statistical software R (version 4.3.3) was used to analyze and visualize data (11). To summarize the characteristics of the cohort, we used descriptive statistics using absolute and relative frequencies for categorical variables and median and interquartile range for quantitative variables. To compare the categorical variables among two or more independent groups, chi-square test or Fisher’s exact test (when appropriate) was used. For the continuous variables, in particular, for anti-integrin αvβ6 IgG, normality was verified both graphically (through quantile–quantile plots and histograms; see **Supplementary Figure S1**) and with the Shapiro–Wilk test. Since normality was rejected, we used Mann–Whitney *U*-test for two-group comparisons and Kruskal–Wallis test with *post-hoc* Holm’s correction for comparisons with more than two groups. The correlations between variables were analyzed with Spearman’s rank test.

The anti-integrin αvβ6 IgG was tested as a continuous variable and as a binary variable. The cutoff for anti-integrin αvβ6 IgG as a binary variable was initially set to a value greater than the value of the controls (mean of non-IBD patients + 3 standard deviations). To evaluate the diagnostic performance of anti-integrin αvβ6 IgG of UC in our cohort, a receiver operating characteristics curve (ROC) analysis was performed, and the area under the curve (AUC) and its 95% confidence interval (CI) were estimated (R package “pROC”) (12). Based on the ROC curve, the optimal cutoff for maximizing the sensitivity and specificity of anti-integrin αvβ6 IgG test was chosen based on the top-left point and Youden’s methods. Lastly, the composite outcome of hospitalization and colectomy were analyzed in patients with a follow-up of at least 1 year after sampling. The data were visualized with Kaplan–Meier graph and analyzed with log-rank test up to 5 years after sampling (R package “Survival”) (13).

## 3 Results

### 3.1 Patients’ population

We enrolled a total of 228 patients from the two clinical centers. This cohort included 37 controls and 34 IBS, 50 CD, and 107 UC patients, respectively. The UC patients from the two centers presented similar disease duration, age at sampling, and localization according to Montreal classification, but with a different percentage of male patients (Torino, To, 47% vs. Treviso, Tv, 68%). The patients from Torino presented more frequently with active disease (i.e., PMS >2: To 89% vs. Tv 30%) and ongoing steroid treatment (To 78% vs. Tv 7%). On the other hand, less patients were receiving advanced therapies (To 11% vs. Tv 64%). Anyway, the values of anti-integrin αvβ6 IgG of UC patients presented no significant differences between the two centers (Mann–Whitney *U*-test: *p* = 0.62). Therefore we proceeded on analyzing the group of UC patients of the two centers as a single group of patients (**Supplementary Table S1**). The 37 controls included 13 healthy donors and 24 diseased controls with other medical conditions, including celiac disease and various autoimmune diseases (see **Supplementary Table S2**). The characteristics of UC and CD patients are summarized in **Table 1**. The median age at sampling was 45 (IQR 28–55) years in UC and 32.5 (IQR 21–51) in CD (*p* = 0.009). Moreover, five pediatric UC and nine pediatric CD patients were included. The disease extension of UC patients was proctitis (E1) in nine (8.4%), left side colitis (E2) in 39 (36%), and pancolitis (E3) in 59 (55%) patients. The UC patients presented active disease in 54 (50%) cases and were receiving steroid or advanced therapies at time of sampling in 34% and 46% of cases, respectively. The CD patients presented ileal involvement in 15 (30%) cases, colonic in 10 (20%), and ileocolonic involvement in 25 (50%). They were receiving steroid or advanced therapies at the time of sampling in 2% and 82% of cases, respectively.

**Table 1:** Characteristics of the Ulcerative colitis (UC) and Crohn’s disease (CD) patients.

Patients' characteristics	Ulcerative colitis (*N* = 107, +%)	Crohn’s disease (*N* = 50, +%)	*p*
Age at sampling (years)	45 (IQR 28–55)	32.5 (IQR 21–51)	0.009
Pediatric (age at sampling <18 years)	5 (4.7%)	9 (18%)	0.013
Age at diagnosis	31 (IQR 22–45)	25.5 (IQR 18.3–30.8)	0.040
Disease duration	8 (IQR 2–15)	4 (IQR 2–9)	0.001
Sex (M)	62 (58%)	29 (58%)	1.000
Smoking status			0.061
Never smoker	69 (64%)	36 (72%)	
Active smoker	9 (8.4%)	8 (16%)	
Former smoker	29 (27%)	6 (12%)	
Montreal classification (UC)			
Extension			
E1	9 (8.4%)	NA	
E2	39 (36%)	NA	
E3	59 (55%)	NA	NA
Disease activity (UC)			
PMS	2 (IQR 0–4)	NA	
PMS (≥2)	54 (50%)	NA	NA
Montreal classification (CD)			
Age			
A1	NA	11 (22%)	
A2	NA	29 (58%)	
A3	NA	10 (20%)	NA
Location			
L1	NA	15 (30%)	
L2	NA	10 (20%)	
L3	NA	25 (50%)	NA
Behavior			
B1	NA	28 (56%)	
B2	NA	14 (28%)	
B3	NA	8 (16%)	NA
Perianal disease	NA	17 (34%)	NA
Treatment at the time of sampling			
Steroid	34 (32%)	1 (2%)	<0.001
Mesalazine	90 (84%)	12 (24%)	<0.001
Immunosuppressive	12 (11%)	1 (2%)	
Advanced therapies^a^	49 (46%)	41 (82%)	

Continuous variables are expressed as medians (and interquartile ranges) and categorical variables as absolute frequencies and percentages. Chi-square test or Fisher’s exact test, when appropriate, was used to compare categorical variables, and Mann–Whitney *U*-test was used for the continuous variables.

*N*, total number; *y*, years; IQR, interquartile range; M, male; PMS, partial Mayo score; NA, not applicable.

a Advanced therapies include anti-TNF (tumor necrosis factor), vedolizumab, ustekinumab, Janus kinase (JAK) inhibitors or combination therapies with biologics + immunosuppressant.

**Figure 1:**
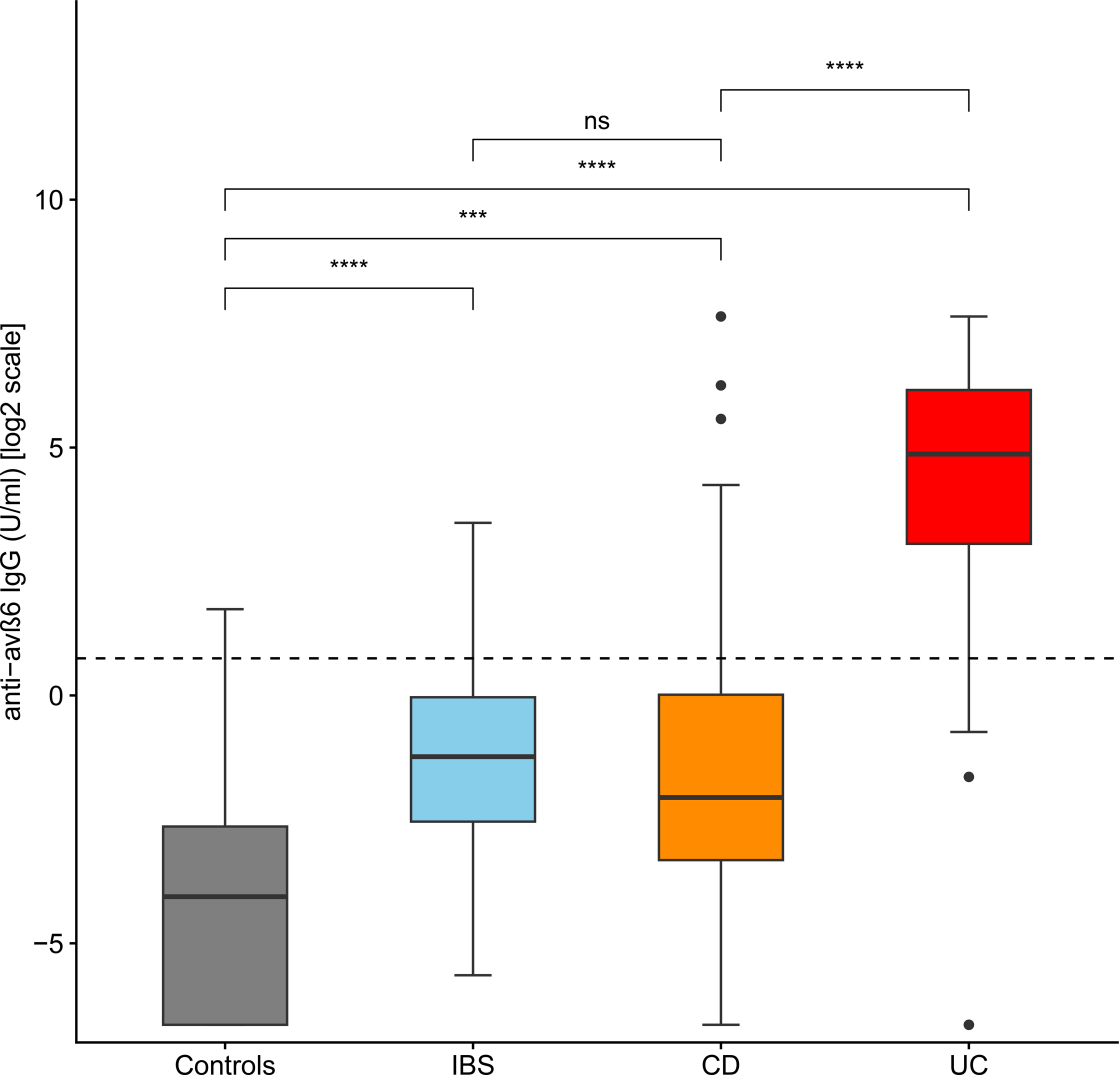
Levels of anti-integrin αvβ6 IgG antibodies in the different groups of patients. The levels of anti-integrin αvβ6 IgG antibodies are displayed in base 2 logarithmic scale (log2). The *p*-values were computed with *post-hoc* Dunn’s test. The dashed line represents the optimal cutoff to distinguish ulcerative colitis cases from other patients. IBS, irritable bowel disease; CD, Crohn’s disease; ns, not significant; ***p < 0.001; ****p < 0.0001.

The median values of anti-integrin αvβ6 IgG in controls, IBS, CD, and UC were 0.06 U/mL (IQR 0–0.16), 0.43 U/mL (IQR 0.17–0.98), 0.24 U/mL (IQR 0.1–1.01), and 29.2 U/mL (IQR 8.3–71.6), respectively. The values of anti-integrin αvβ6 IgG were higher in UC than in the other groups (*post-hoc* Holm’s correction: *p* < 0.001 vs. controls, IBS and CD). CD and IBS presented higher values compared to controls (*post-hoc* Holm’s correction: *p* = 0.002 and *p* = 0.001, respectively). On the other hand, the difference between CD and IBS did not reach statistical significance (*post-hoc* Holm's correction: p=0.277) (see **Table 2**, **Figure 1**, and **Supplementary Figure S2**).

**Table 2:** Cohort composition and anti-integrin αvβ6 IgG comparison among groups.

Group	*N* (*N* = 228)	Anti-αvβ6 (U/mL) median (IQR, range)	Kruskal–Wallis test	UC vs. others (adjusted *p*-value)	CD vs. others (adjusted *p*-value)	IBS vs. others (adjusted *p*-value)
Controls^a^	37	0.06 (IQR 0–0.16, range 0–3.34)	**<0.001**	**<0.001**	**0.002**	**0.001**
IBS	34	0.43 (IQR 0.17–0.98, range 0.02–11)		**<0.001**	0.277	NA
CD	50	0.24 (IQR 0.10–1.01, range 0–200)		**<0.001**	NA	0.277
UC	107	29.2 (IQR 8.3–71.6, range 0–200)		NA	**<0.001**	**<0.001**

The difference among groups was tested with Kruskal–Wallis test and *post-hoc* Holm’s correction for multiple testing. In bold are the significant *p*-values below <0.05.

IBS, irritable bowel disease; CD, Crohn’s disease; UC, ulcerative colitis; IQR, interquartile range; NA, not applicable.

a Controls included 13 healthy donors and 24 diseased controls (for details, see **Supplementary Table S1**).

### 3.2 Cutoff of positivity

**Figure 2:**
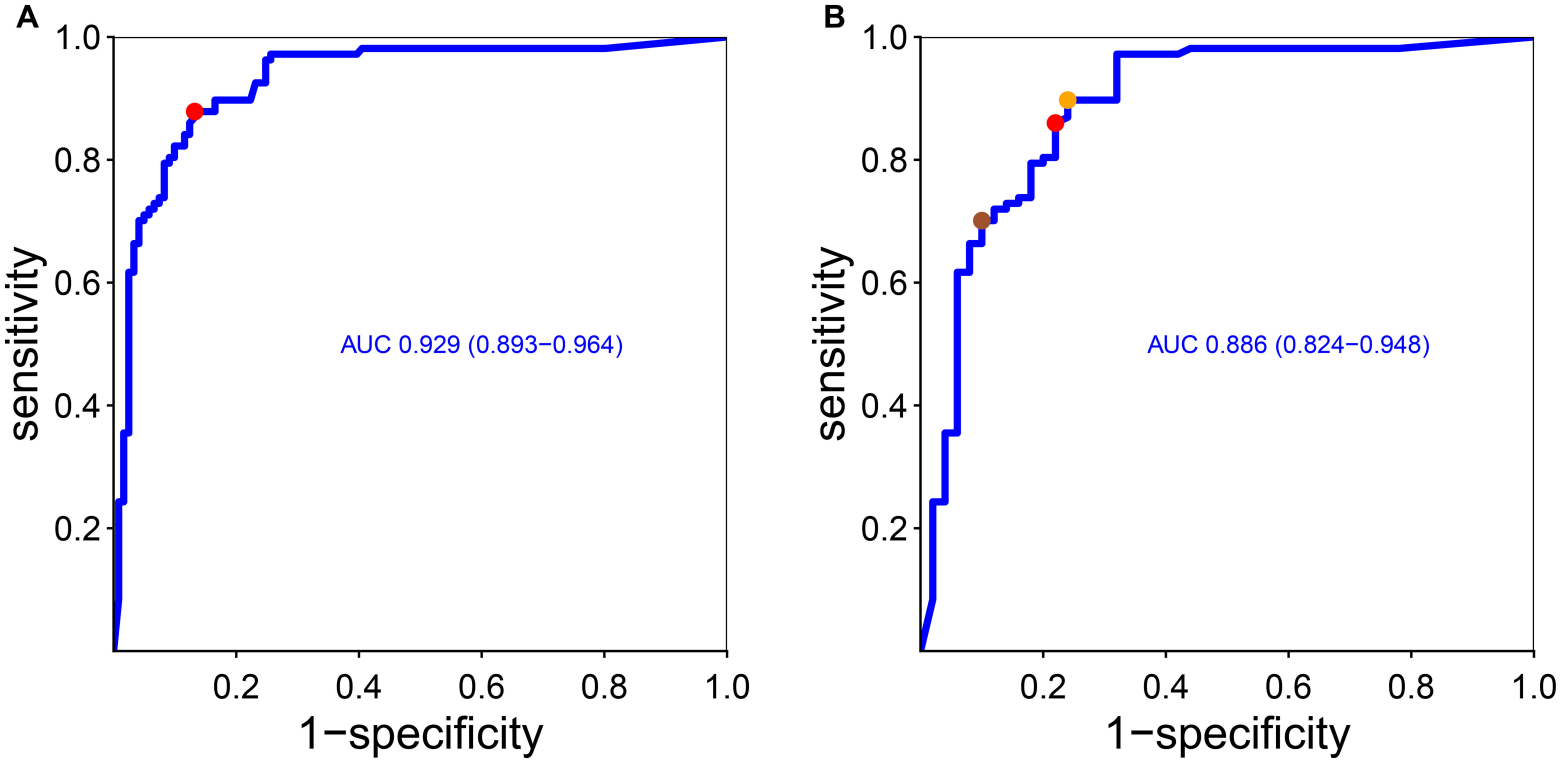
Receiver operating curve of anti-integrin αvβ6 IgG antibodies and diagnosis of ulcerative colitis considering other conditions **(A)** and only Crohn’s disease **(B)**. As stated in the “Methods” section, we found the optimal cutoff that maximized sensitivity and specificity according to Youden’s point and the top-left point method. In **(A)**, Youden’s point and the top-left point correspond to the same value (red dot, corresponding to 1.68 U/mL). In **(B)**, the red dot and the orange dot represent the closest top-left point and Youden’s point, respectively. The sienna dot represents a threshold that maximized the specificity (see the main text). AUC, area under the curve (and 95% confidence interval).

The cutoff of positivity of anti-integrin αvβ6 IgG, based on the values of the non-IBD patients, was set to 4.93 U/mL but rounded up to 5 U/mL (corresponding to the mean of non-IBD patients + 3 standard deviations). This threshold identified correctly 85 UC patients (79%) with very high specificity versus non-IBD (98.5%) and lower versus CD (80%). A ROC curve analysis was used to find the optimal threshold that maximized the sensitivity and specificity of anti-integrin αvβ6 IgG antibodies in identifying UC compared to the other groups of patients. The area under the curve (AUC) was 0.93 (95% C.I. 0.89–0.96; see **Figure 2**). The best value to classify optimally UC according to our ROC curve (with Youden’s and closest top-left point methods) was estimated in 1.68 U/mL, with sensitivity of 87.9% (95% C.I. 82–93) and specificity of 86.8% (95% C.I. 80–93) compared to non-UC patients with specificity of 94.4% for non-IBD (including IBS) and 76% for CD.

Next, we wanted to find the anti-integrin αvβ6 IgG value that best discriminates UC from CD in order to help in the diagnosis in case of unclassified IBD (IBD-U). The ROC curve showed an AUC of 0.89 (C.I. 0.82–0.95). The optimal cutoffs according to Youden’s and closest top-left point methods were, respectively, 1.12 U/mL, with sensitivity of 89% (95% C.I. 84–96) and specificity of 76% (95% C.I. 64–88), and 2.12, with sensitivity of 86% (95% C.I. 79–92) and specificity of 78% (95% C.I. 66–88). In clinical practice, it would be useful to have a threshold that maximizes the specificity for UC versus CD to help classify IBD-U patients, with repercussions for medical treatment options and surgical options. Therefore, we opted to set a higher threshold that maximized the specificity for UC over CD: therefore, the best value to distinguish UC from CD would be 13 U/mL, with specificity of 90% (95% C.I. 82–98) and a sensitivity of 70% (95% C.I: 61–79).

### 3.3 Pediatric population

In our study, we included five pediatric UC patients and nine CD patients. The UC patients presented a median value of anti-integrin αvβ6 IgG of 68 U/mL (range, 11–200), whereas for CD it was 0.13 (range, 0–0.95). It is worth noting that all UC patients presented anti-integrin αvβ6 IgG values of antibodies above 2 U/mL and all CD patients below 2 U/mL, allowing complete discrimination in this particular setting (**Supplementary Figure S3**).

### 3.4 Disease location

**Figure 3:**
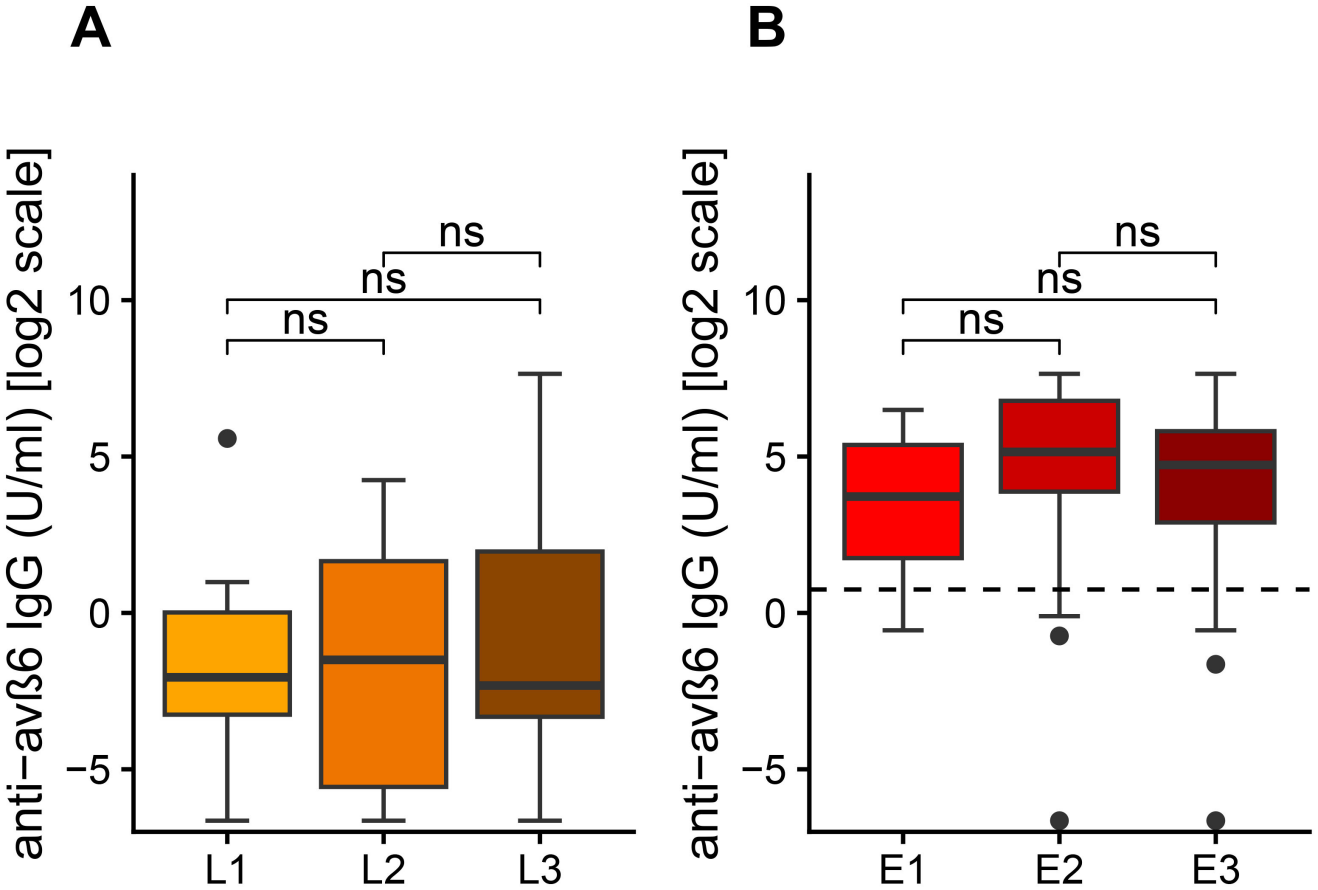
Levels of anti-αvβ6 IgG antibodies based on disease location according to Montreal classification **(A)** in Crohn’s disease (CD) and in ulcerative colitis (UC). The levels of anti-integrin αvβ6 IgG antibodies are displayed in base 2 logarithmic scale (log2). The *p*-values were computed with *post-hoc* Dunn’s test. L1, ileal involvement; L2, colonic involvement; L3, ileo-colonic involvement; E1, proctitis; E2, left side colitis; E3, pancolitis; ns, not significant.

We then analyzed the differences of anti-integrin αvβ6 IgG in patients with UC and CD according to Montreal classification of disease location. We did not find statistical differences of anti-integrin αvβ6 IgG in UC patients according to disease localization (Kruskal–Wallis test: *p* = 0.15). However, we noted that patients with very high values of anti-integrin αvβ6 IgG (>100 U/mL) present either left colitis (E2) or pancolitis (E3) and never with proctitis (**Figure 3A**). Similarly, in CD patients, we did not observe any statistical difference of anti-integrin αvβ6 IgG based on disease localization (Kruskal–Wallis test: *p* = 0.90). Among the 11 CD patients with values greater than 2 U/mL, 10 patients presented either a colonic (L2) or an ileocolonic involvement (L3) (**Figure 3B**).

### 3.5 Correlations and associations in UC

**Figure 4:**
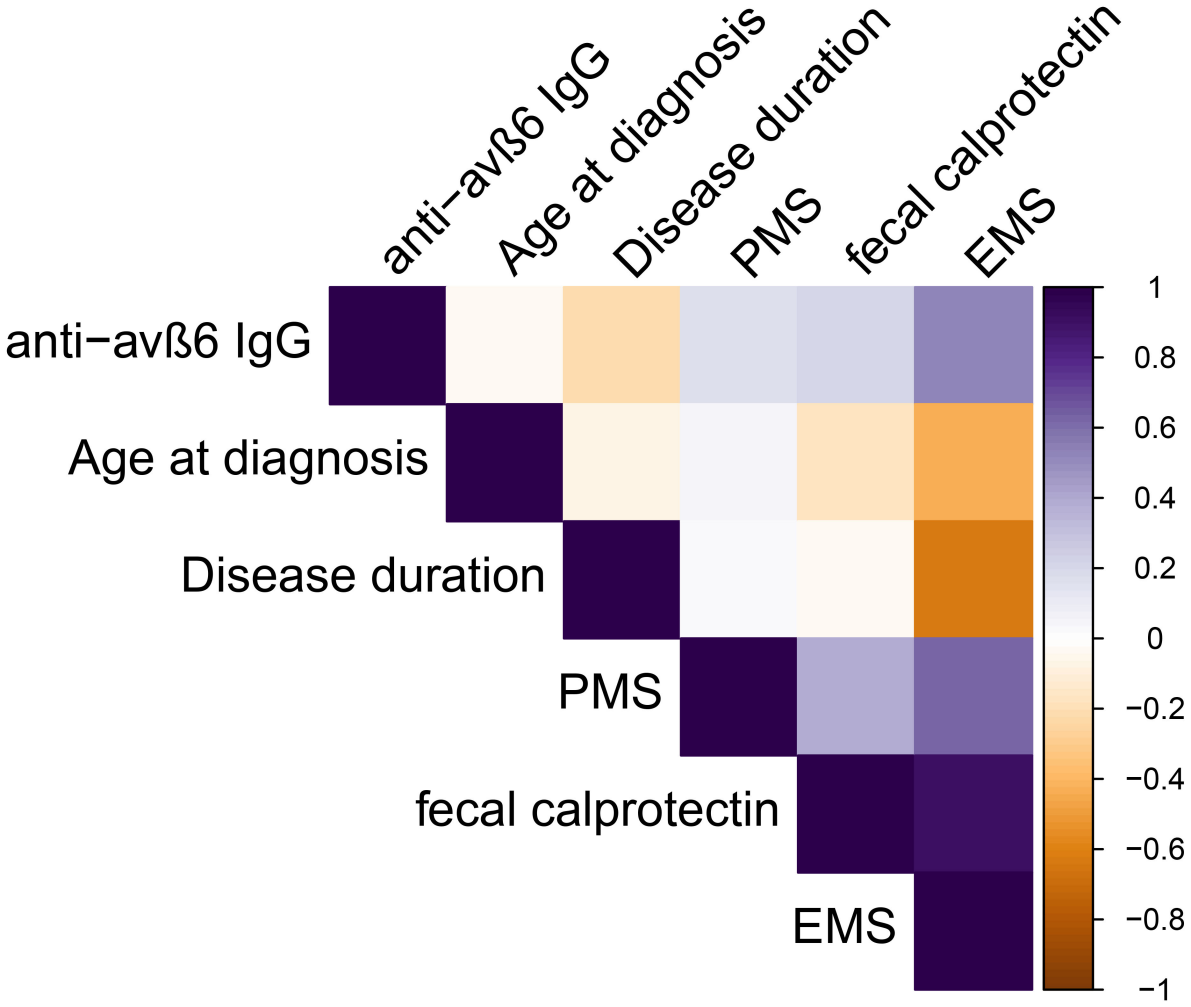
Spearman’s correlations among continuous variables analyzed in ulcerative colitis patients. PMS, partial Mayo score, EMS endoscopic Mayo score.

Among UC patients, the anti-integrin αvβ6 IgG presented a weak negative correlation with disease duration (*R* = -0.22, *p* = 0.03), but not with age at diagnosis (*R* = 0.08, *p* = 0.42) or partial Mayo score (*R* = 0.12, *p* = 0.24). In a subgroup of patients, data on FCP and colonoscopy were available. The anti-integrin αvβ6 IgG correlated weakly with FCP (*R* = 0.28, *p* = 0.04) and moderately with endoscopic Mayo score (*R* = 0.60, *p* = 0.03) (see **Supplementary Table S3** and **Figure 4**). We found no statistical difference in patients according to smoking status (Kruskal–Wallis test: *p* = 0.09), disease duration of at least 5 years (Mann–Whitney *U*-test: *p* = 0.062), or with active clinical disease, defined as PMS ≥2 (*p* = 0.58) (**Supplementary Figure S4**). Then, we compared the antibody levels among different treatment groups of patients. The values of anti-integrin αvβ6 IgG presented similar values in patients on treatment with steroid compared to others (Mann–Whitney *U*-test: *p* = 0.72) and in patients on advanced therapies (including biological drugs) compared to immunosuppressive alone or mesalazine treatment (Kruskal–Wallis test: *p* = 0.76) (**Supplementary Figure S5**).

### 3.6 Predictive outcomes

**Figure 5:**
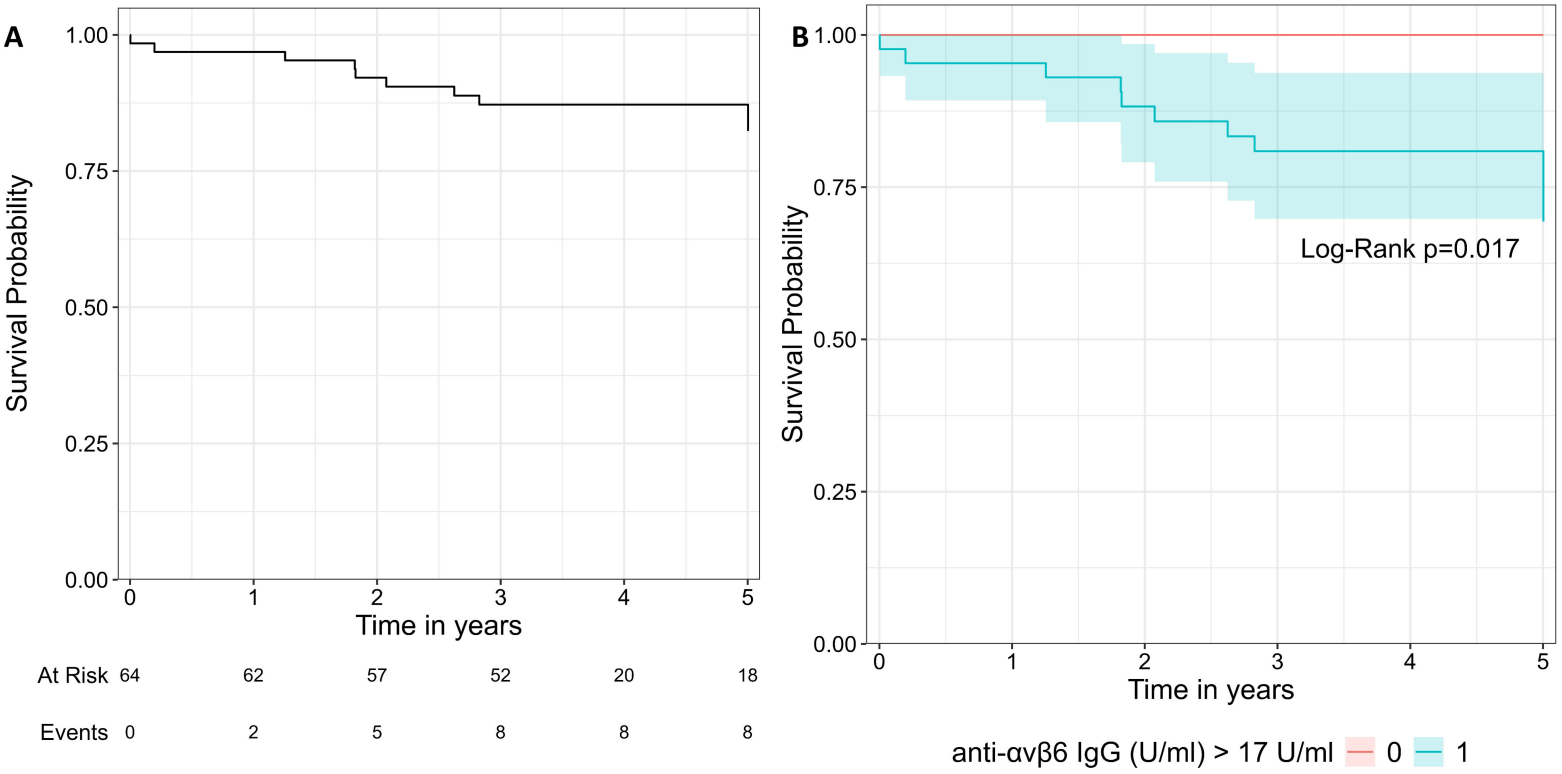
Survival curve of the composite outcome including hospitalization and colectomy. **(A)** represents the whole cohort. In **(B)**, the cohort is split in two groups with the threshold of 17 U/mL. The two groups were compared with log-rank test.

Lastly, we performed an exploratory analysis in UC patients with a follow-up at least of 1 year after the sampling (*N* = 64). We considered the composite outcome of hospitalization and colectomy as event. We noted that all patients that reached the composite outcome (*N* = 9) presented values of anti-integrin αvβ6 IgG higher than 17 U/mL and a disease extension E2–E3. Using the threshold of 17 U/mL, the log-rank test showed a significant difference between the two groups (*p* = 0.02, **Figure 5**). We did not perform Cox regression analysis due to the limited number of patients and events.

## 4 Discussion

In our multicentric observational study involving two Italian centers, patients with IBD and various controls groups were enrolled, including healthy donors and patients with autoimmune diseases, celiac disease, and IBS. In our study, we could validate the optimal performance of anti-integrin αvβ6 IgG in identifying UC patients with an AUC of 0.93 and set the threshold to a cutoff of 1.68 U/mL with very good sensitivity (87.9%) and good specificity to non-UC patients (86.8%). The sensitivity for UC is slightly lower compared to the original description of the anti-integrin αvβ6 IgG (92%) (6), but it is higher than in Swedish and Italian cohorts (76% and 52%, respectively) (7, 9). Intriguingly, a recent multicentric Japanese study including 1,241 UC, 796 CD, and 206 controls with other gastrointestinal conditions found a very similar threshold of positivity for anti-integrin αvβ6 IgG (1.64 U/mL) and with strikingly similar results of sensitivity for UC at 87% and specificity at 82% for CD and 88% for other gastrointestinal disorders (14). We could confirm the optimal specificity of anti-integrin αvβ6 IgG toward healthy donors and non-IBD conditions (94.4%), including different rheumatological disorders, celiac disease, and IBS (6, 7, 15). A recent work confirmed that anti-integrin αvβ6 IgG performed better than calprotectin and C-reactive protein (CRP) in distinguishing UC from diseased controls (including IBS, celiac disease, and other conditions). In the same publication, the diagnostic performance of anti-integrin αvβ6 IgG could be further increased by combining anti-integrin αvβ6 IgG and calprotectin but not CRP (16). Considering the low number of controls with measured calprotectin at the time of sampling, we did not perform this analysis in our cohort.

Anti-integrin αvβ6 IgG presented a lower specificity for UC versus CD. In our cohort, 11 adult CD patients presented positive antibodies. These patients presented more frequently with colonic involvement (L2-3). False positive results in CD have been reported in 17%–21% (6, 8, 13) and up to 32% pediatric CD patients (15). Colonic involvement has been described as the only independent factor associated with false positive results in CD (14). Detectable anti-integrin αvβ6 IgG in CD has been associated with more frequent endoscopic and pathologic UC-like colitis (15). Intriguingly, a subgroup (31%) of patients with immune checkpoint inhibitor-induced colitis (ICI-colitis) presented anti-integrin αvβ6 IgG. This was associated with endoscopic UC-like characteristics, grade 3 colitis, and steroid resistance (17). All things considered, these observations make us speculate that anti-integrin αvβ6 IgG antibodies are associated with a specific pattern of colonic mucosa inflammation resembling UC rather than be unique for UC. However, anti-integrin αvβ6 IgG antibodies have also been shown not only in patients with primary sclerosing cholangitis (PSC) with IBD (92%) but also without IBD (74%) (18). The rationale of their serological presence in the case of liver disease may be found in the selective expression of integrin αvβ6 in the disease-associated epithelia of both bile duct and colon (18, 19). Such data were not confirmed in all reports: more recent publications indicate a lower positivity of anti-integrin αvβ6 autoantibodies in patients with PSC without IBD (39%) (19) or even comparable levels to controls (20). In our cohort, unfortunately there were no patients with PSC, so we cannot significantly contribute to this debate.

In clinical practice, the utility of anti-integrin αvβ6 IgG antibodies in differentiating UC vs. CD is of arguable importance considering that endoscopy (and histology) is generally sufficient to separate UC from CD. We think that the most important role of anti-integrin αvβ6 IgG antibodies would be in helping classify the patients with IBD-U, where endoscopy and histology have not definitively discriminated the two entities (4). Therefore, every attempt to classify IBD-U should be prompted, considering the implications on medical treatments and surgical options—for instance, colectomy is considered a definitive treatment option for UC, whereas sparing surgery is indicated for CD. A false positive may lead to colectomy for the wrong patient with irremediable consequences, so we think that, in this setting, the risk of the wrong classification weights more for false positive than false negative. Therefore, maximizing specificity (i.e., reducing the false positive) is more important than sensitivity. In this regard, we weighted our analysis to find a higher threshold of positivity to distinguish UC from CD. Here we propose an optimal threshold of 13 U/mL to confirm a UC diagnosis versus CD with specificity of 90% and sensitivity of 70%. This test performance is better than atypical p-ANCA or ASCA in discriminating the two main forms of IBD (5) despite the fact that we could not directly compare the performance of these two markers in our cohort. Thus, we propose a high cut-off to improve the ability to distinguish UC from CD, especially in patients with IBD-U. Prospective studies on IBD-U patients are needed to confirm if this strategy may be applied in this context and determine if the disease course differs based on the presence of detectable antibodies. This work may pave the way for a prospective study aiming at finding a clinical score integrating endoscopic, histological, and serological data to reduce the cases of IBD-U. A recent publication showed indeed that most IBD-U patients do have detectable antibodies (16). We also analyzed the possible correlation of anti-integrin αvβ6 levels with UC characteristics. We observed that anti-integrin αvβ6 IgG antibodies presented similar levels when grouping the patients according to disease extension. However, very high levels of anti-integrin αvβ6 IgG antibodies were seen only in patients with colonic involvement (E2–E3) rather than E1 (disease limited to the rectum). The association between the presence of antibodies and the extent of colonic involvement in UC is still debated. While some authors have described this association (6, 10), others (9), including our group, have not confirmed it. Similarly, we found no correlation with partial Mayo score or active disease state (PMS ≥2) similar to the findings of Marafini et al. (9) and opposite to that of Rydell et al. (7) We found a moderate correlation with endoscopic activity and a weak correlation with FCP, albeit data on endoscopic activity were available only in a small group of patients (19). These differences may be related to different recruitment criteria among studies. Our cohort is heterogeneous, and the patients were receiving different treatments at the time of sampling, possibly affecting the analysis. However, we did not observe a statistical difference in patients receiving immunosuppressive or advanced therapies, probably because of the relatively small sample size. In addition, the sampling was rarely performed at the time of diagnosis. Recently, Pertsnidou et al. showed a possible prognostic role of anti-integrin αvβ6 IgG in recently diagnosed UC. The UC patients with positive anti-integrin αvβ6 IgG presented with a more aggressive disease course (i.e., greater disease extent and endoscopic activity). The anti-integrin αvβ6 IgG levels remained stable at 3 months in patients with aggressive disease and decreased significantly in patients with an indolent course of disease (16).

With the limitation of the relatively low number of subjects and events, we explored if anti-integrin αvβ6 IgG could predict the composite outcome of hospitalization and/or colectomy in patients with at least 1 year of follow-up. Indeed all patients reaching the outcome presented levels of anti-integrin αvβ6 above 17 U/mL. The survival curve showed a significant difference between the group of patients with higher (≥17 U/mL) and lower antibodies (<17 U/mL). Livanos et al. showed that higher levels of anti-integrin αvβ6 IgG were associated with a composite outcome that included also a need for biological drugs or switch (10). In our cohort, the patients enrolled in Torino were all candidate to start an advanced therapy at the time of sampling. Therefore, we could not include the need for biological drug in the composite outcome for statistical bias. Thus, larger prospective studies are needed to confirm this observation.

Integrin αvβ6 is restricted to the epithelia and is upregulated during epithelial repairs and tumorigenesis (21). Therefore, it has been suggested that the formation of anti-integrin αvβ6 IgG is secondary to the epithelial colonic damage (6, 17). These antibodies have been demonstrated to inhibit the RGD binding motif, thereby blocking the interaction with several ligands, including fibronectin, in a dose-dependent manner (6, 17). A similar interaction is required for the liberation of transforming growth factor β1 (TGF-β1) from the latent associated peptide (LAP) to exert its function (22). In mouse models, the absence of RGD motif in TGF-β LAP replicated the features of knockout mice for integrin β-6 (*itgb6* ^-/-^) or TGF-β1 (*tgfb1* ^-/-^) (22). Consequently, it can be hypothesized that anti-integrin αvβ6 IgG may diminish TGF-β activation, reducing epithelial–mesenchymal transformation and tissue repair. Nevertheless, the data regarding TGF-β in IBD are somewhat contradictory—for instance, Sedda et al. reported that restoring TGF-β signaling through SMAD-7 inhibition suppresses inflammation in CD patients (23), whereas Ghorbaninejad et al. reported that inhibition of TGF-β signaling suppresses epithelial–mesenchymal transformation and preserves tight junction integrity (24). However, we have to consider that the majority of evidence of the role of TGF-β were collected in CD rather than UC. Furthermore, very-early-onset colitis and brain abnormalities have been described in three families harboring an integrin alpha-v variant (ITGAV) (25) and in a patient with homozygous integrin β-6 polymorphism (ITGB6) (26). The abnormalities resemble the phenotype of TGF signal pathologies (25). Future studies should be conducted to ascertain whether anti-integrin αvβ6 IgG is associated with diminished intestinal TGF-β signaling and to investigate the implication of this association of inflammation and fibrosis.

Uzzan et al. showed a skewed IgG response with a marked increase of IgG-producing short-lived plasma cells in the gut mucosa and peripheral blood of UC patients (8). This, in addition to the evidence of lymphoplasmacytic infiltrate, anti-microbial antibodies, and the protective role of the single nucleotide polymorphism in FCGR2A (rs1801274), which encodes an activating IgG Fcg receptor (FcgR) expressed by myeloid cells, further supports the possible involvement of B cells in UC pathogenesis (27). However, rituximab, an anti-CD20 monoclonal antibody that depletes B cells, was ineffective in treating UC in a small placebo-controlled trial involving patients with refractory disease (28). There is even a study reporting that rituximab increases the risk of IBD (29). Therefore, further studies are needed to explore the role of plasma cells in IBD pathogenesis and the possibility of targeting B cells with other therapies in UC.

Our study presents several limitations. First, this is an observational study, and data on colonoscopy and fecal calprotectin were limited to a subgroup of patients because they were not routinely measured on the same day of sampling. The sampling was performed rarely at the time of diagnosis, and therefore some seronegative UC may have been sampled after years of remission. On the other hand, this allows us to generalize our results to all time points of the patients’ history, also considering that the two centers included IBD patients who were both naïve and experienced with advanced therapies. Finally, other forms of acute colitis (such as infectious or diverticular disease) were not included.

In conclusion, this study validates the “anti-integrin αvβ6 ELISA kit” and confirms the diagnostic role of anti-integrin αvβ6 IgG in UC with an optimal cutoff of 1.68 U/mL and with good sensitivity and optimal specificity compared to the controls. The presence of antibodies supports the diagnosis of UC over CD. However, a group of CD patients with colonic involvement may present positive antibodies. Therefore, we propose a higher threshold of 13 U/mL to maximize the specificity for UC over CD. This threshold may be helpful to reduce unclassified colitis (IBD-U). Further studies are needed to build composite scores integrating endoscopic and serological data to obtain the best results. The availability of a commercial ELISA kit will allow the inclusion of anti-integrin αvβ6 IgG assessment in the routine diagnostic algorithm for IBD in Europe. Further studies are needed to evaluate longitudinally the levels of anti-integrin αvβ6 IgG and explore if these antibodies correlate with clinical and/or endoscopic activity of disease and the possible influence of specific treatments on the presence of antibodies. If the pathogenic role of anti-integrin αvβ6 IgG in blocking mucosal healing in UC patients was confirmed, potential therapies targeting TGF-β and/or mucosal IgG-producing plasma cells could be explored in the future.

## Data Availability

The raw data supporting the conclusions of this article will be made available by the authors, under justified request to the Corresponding Author.
