# Supplementary material for: Antibodies against integrin αvβ6 have high diagnostic accuracy for ulcerative colitis
Source: Front Immunol. 2025 Aug 21;16:1641329. doi: 10.3389/fimmu.2025.1641329 (PMC12408611; doi:10.3389/fimmu.2025.1641329)
Supplement: Supplementary file 1 [file DataSheet1.docx]

**SUPPLEMENTARY TABLES**

**Supplementary table S1. Characteristics of the UC patients in the two Centers.**

|  | **Treviso**  **(N=70)** | **Torino**  **(N=37)** |
| --- | --- | --- |
| Age at sampling (y) | 45 (IQR 27-52) | 45 (IQR 31-64) |
| Pediatric (N) | 5 | 0 |
| Age at diagnosis (y) | 30 (IQR 20-42) | 36 (IQR 23-50) |
| Disease duration (y) | 7 (IQR 2-15) | 8 (IQR 2-15) |
| Sex (M) | 37 (47%) | 25 (68%) |
| Localization |  |  |
| E1 | 5 (7.1%) | 4 (11%) |
| E2 | 26 (37%) | 13 (35%) |
| E3 | 39 (58%) | 23 (54%) |
| Disease activity |  |  |
| PMS | 1 (IQR 0-2) | 4 (IQR 3-5) |
| PMS (>=2) | 21 (30%) | 33 (89%) |
| Treatment at time of sampling |  |  |
| Mesalazine | 59 (84%) | 31 (84%) |
| Steroid | 5 (7%) | 29 (78%) |
| Immunesuppressive | 4 (5.7%) | 8 (22%) |
| Advanced therapies* | 45 (64%) | 4 (11%) |
| Anti-integrin αvβ6 IgG | 33 (IQR 8.8-200) | 24 (IQR 8.6-200) |

The continuous variables are depicted as median and interquartile range. The categorical variables as absolute values and percentage. N=total number. y=year. IQR=interquartile range. M=male; PMS=Partial Mayo Score. * Advanced therapies include anti-TNF (Infliximab, adalimumab, golimumab), Vedolizumab, combination therapies (anti-TNF/Vedolizumab + immunosuppressive drug), small molecules (filgotinib or tofacitinib).

**Supplementary table S2. Diagnosis of control group**

| **Controls** | **N=37** |
| --- | --- |
| Healthy donors | 13 |
| Celiac disease | 2 |
| Rheumatoid arthritis | 5 |
| Psoriasis/Psoriatic arthritis | 8 |
| Pemphigus | 2 |
| Rheumatic polymyalgia | 2 |
| Overlap syndrome/MCTD | 3 |
| Other rheumatic conditions:   - HLA-B27 Arthritis - Gout | 2 |

The absolute frequency of patients is reported. MCTD=Mixed connective tissue disease.

**Supplementary table 3. Correlations between anti-integrin αvβ6 IgG** **and clinical characteristics of UC patients**

|  | **N** | **R** | **p** |
| --- | --- | --- | --- |
| **Age at diagnosis** | 107 | -0.079 | 0.417 |
| **Disease duration** | 107 | **-0.217** | **0.025** |
| **PMS** | 107 | 0.115 | 0.238 |
| **Fecal calprotectin** | 55 | **0.284** | **0.036** |
| **EMS** | 14 | **0.595** | **0.025** |

The Spearman’s correlation rank test was used to assess correlations among Anti-αvβ6 and different disease characteristics. N=patients for whom the data are available. In bold the significant p-values. Abbreviations: PMS=Partial Mayo Score; Endoscopic Mayo Score (EMS).
