# Supplementary figures and images for: Antibodies against integrin αvβ6 have high diagnostic accuracy for ulcerative colitis
Source: Front Immunol. 2025 Aug 21;16:1641329. doi: 10.3389/fimmu.2025.1641329 (PMC12408611; doi:10.3389/fimmu.2025.1641329)

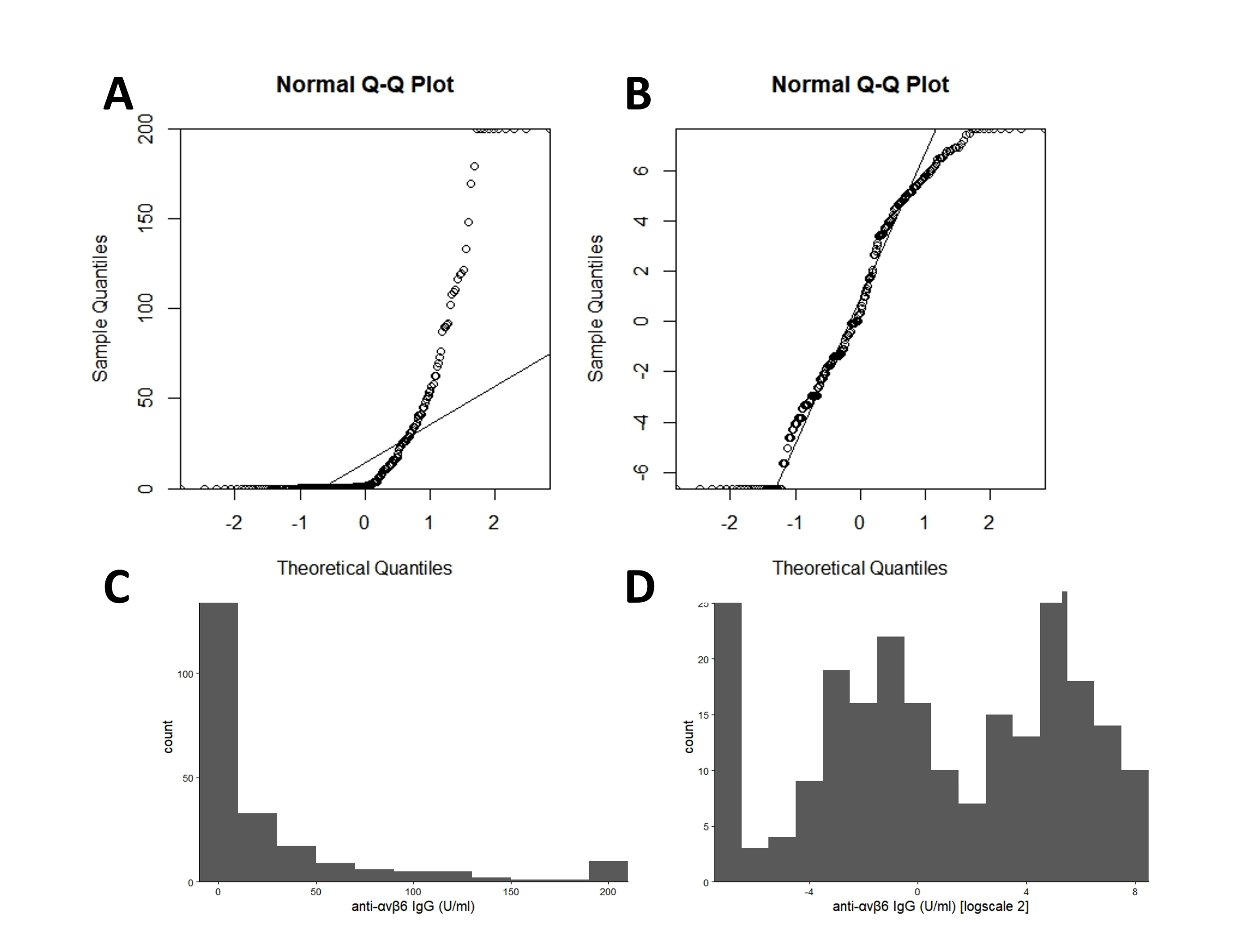

Supplement: Supplementary file 2 [file Image1.png]

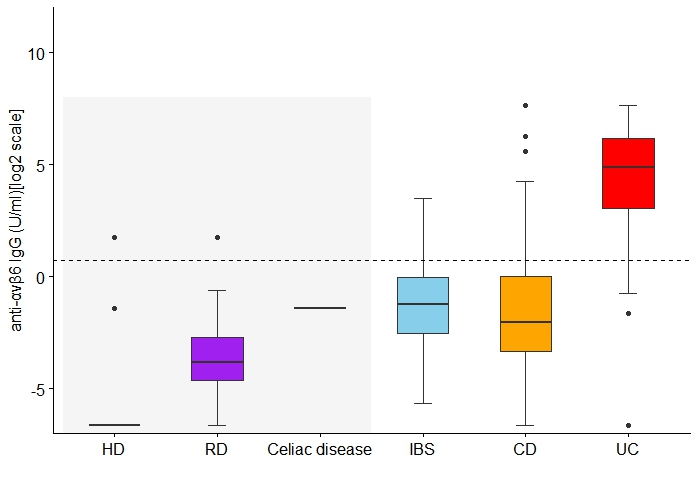

Supplement: Supplementary file 3 [file Image2.jpeg]

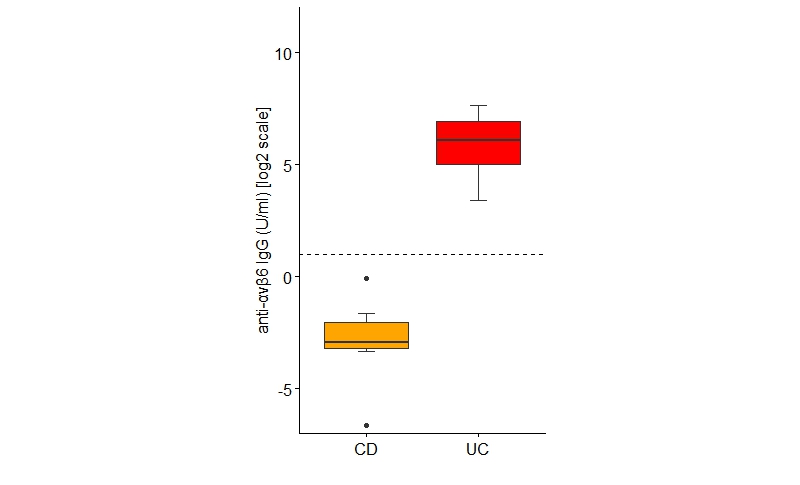

Supplement: Supplementary file 4 [file Image3.jpeg]

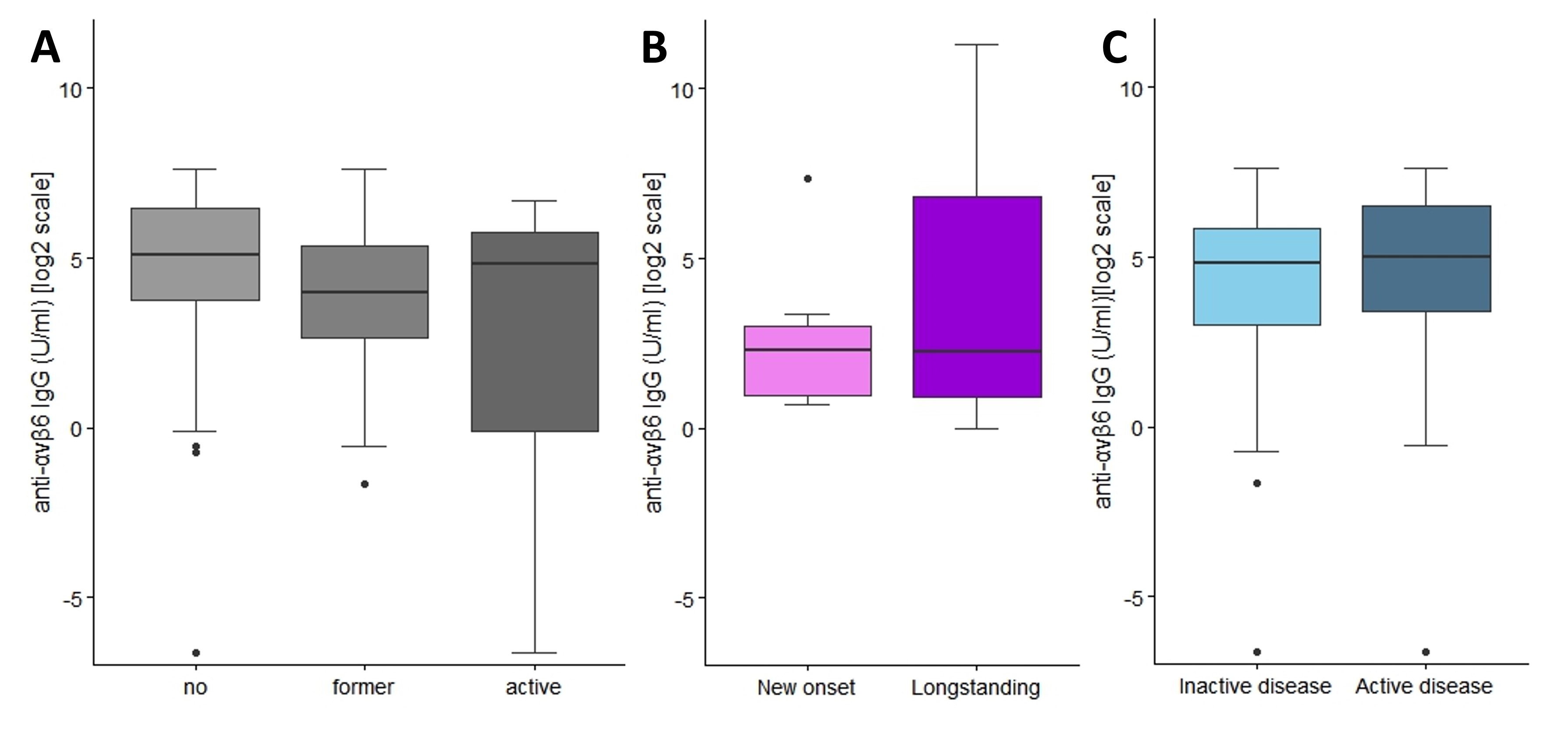

Supplement: Supplementary file 5 [file Image4.jpeg]

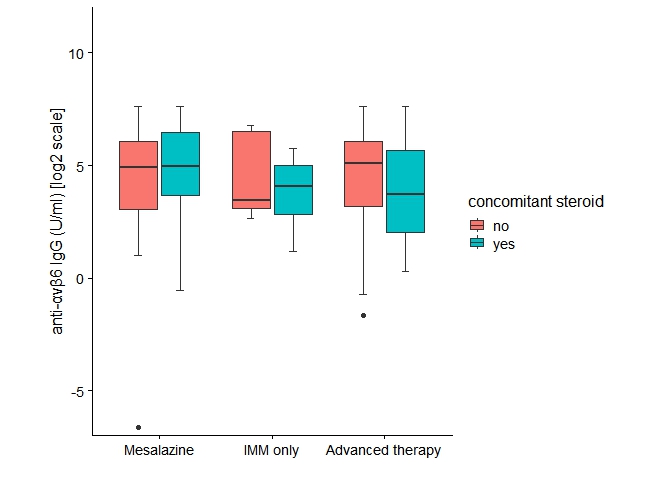

Supplement: Supplementary file 6 [file Image5.jpeg]
